# Efficacy and Safety of Balloon Dilatation of the Eustachian Tube in Children With Eustachian Tube Dysfunction: A Systematic Review and Meta-Analysis

**DOI:** 10.7759/cureus.109250

**Published:** 2026-05-20

**Authors:** Zainab Zujaj, Muhammad Shiraz Yousaf Siddiqui, Ayesha Mansoor, Dipak Chaulagain, Muhammad Shoaib Shahid, Usama Assad-Ullah Maurice Nonnenmacher, Saima Saeed, Nneoma Sam-Odum, Muhammad Abdullah Farooq Cheema, Zeeshan Alvi, Mandeep Kumar

**Affiliations:** 1 Medicine, Al Nafees Medical College and Hospital, Islamabad, PAK; 2 Medicine, Islamic International Medical College, Islamabad, PAK; 3 Special Pathology/Community Medicine/ENT/Opthalmology, Faisalabad Medical University and Allied Hospital, Faisalabad, PAK; 4 Neurosurgery, Uzhhorod National University, Uzhhorod, UKR; 5 Morphology, Jalal-Abad International University, Jalal-Abad, KGZ; 6 Otolaryngology, Faisalabad Medical University and Allied Hospital, Faisalabad, PAK; 7 Pediatrics, Border Health Services, Ministry of National Health Services, Regulations and Coordination, Rawalpindi, PAK; 8 Otolaryngology, Rivers State University Teaching Hospital, Port Harcourt, NGA; 9 Medicine and Surgery, Jalal-Abad State University named after B.Osmonov Medical Faculty, Jalal-Abad, KGZ; 10 Medicine, York St John University, York, GBR; 11 Medicine, Jinnah Medical and Dental College, Karachi, PAK

**Keywords:** balloon dilatation, eustachian tube dysfunction, meta-analysis, otitis media, pediatric, safety, treatment outcomes, tympanogram

## Abstract

The meta-analysis and systematic review evaluated the efficacy and safety of balloon dilatation of the eustachian tube (BDET) in children with eustachian tube dysfunction. The PubMed, Cochrane Library, and ScienceDirect databases were searched in depth to identify relevant studies, and eight retrospective cohort studies were selected. A total of 652 patients were included in these studies. There were no serious complications, and only minor, self-limiting adverse events were seen, the most common of which were epistaxis and a patulous eustachian tube. The primary result was an improvement in the type of tympanogram, and the secondary results were the risk of failure and complications. The pooled analysis of seven studies showed that 64% (95%CI: 50-78%) of cases demonstrated improvement in tympanogram following BDET. However, heterogeneity was high (I² = 90.7%). The pooled failure rate, based on four studies, was 13% (95%CI: 1-17%). Although the evidence on the efficacy and safety of BDET in pediatric patients is encouraging, the overall quality of evidence is limited by the retrospective nature of the included studies, the absence of control groups, and inconsistent outcome measures. These results indicate that BDET may be a useful, minimally invasive procedure for treating eustachian tube dysfunction in children. However, more specific randomized controlled trials with standardized outcome measures are needed to clarify the treatment's long-term effectiveness and its role in clinical practice.

## Introduction and background

Eustachian tube dysfunction (ETD) disrupts middle ear ventilation and pressure, leading to hearing loss and common otitis media infections that worsen functional health and quality of life, especially in children [1-3]. ETD is particularly vulnerable among children, who have less developed tubes both anatomically and functionally, shorter, more horizontally oriented tubes with less effective mechanisms of opening than adults, which can lead to middle ear disease and inefficient ventilation [4]. Persistent ETD may result in chronic otitis media with effusion and permanent hearing loss. The condition also impacts the development of speech and cognitive skills in patients [5,6].

The conventional treatment for pediatric ETD is medical treatment with intranasal corticosteroids and surgical treatment with tympanostomy tube insertion [7,8]. Though such measures might be useful for relieving symptoms in the short run, they do not effectively address the pathology of the eustachian tube. They are commonly associated with procedure-related recurrence or complications [2,9]. Balloon dilatation of the eustachian tube (BDET) has become a minimally invasive procedure over recent years, which enhances eustachian tube and middle ear ventilation [10].

As much as BDET has demonstrated significant outcomes in adult populations, its use in children has not been without controversy because of the paucity of high-quality evidence, inconsistent study designs, and its implications for safety and long-term outcomes in the pediatric population [11]. Several observational studies and small clinical trials have reported positive results, but the overall efficacy and safety profile of BDET in children with ETD remains uncertain [12,13].

Despite the availability of previous systematic reviews, many have included mixed adult and pediatric populations or provided primarily narrative summaries without robust quantitative synthesis specific to children. Additionally, heterogeneity in study design and variability in outcome reporting have limited the applicability of existing findings to pediatric patients. Therefore, there remains a need for an updated, pediatric-focused meta-analysis that provides pooled estimates of clinical outcomes and safety.

In this context, the present systematic review and meta-analysis aims to evaluate the effectiveness and safety of BDET exclusively in children with ETD. By quantitatively synthesizing available evidence on tympanogram improvement, failure rates, and complications, this study seeks to provide more precise and clinically relevant estimates to inform decision-making and guide future research.

## Review

Methodology

Preferred Reporting Items for Systematic reviews and Meta-Analyses (PRISMA) guidelines were followed for this systematic review and meta-analysis, and the study protocol was registered on the International Prospective Register of Systematic Reviews (PROSPERO) (CRD420261341505) [14].

Search Strategy

We used three major databases: PubMed, The Cochrane Library, and ScienceDirect. Various relevant keywords, such as eustachian tube, eustachian tube dysfunction, balloon dilatation, eustachian tube balloon dilatation, BDET, and tuboplasty, were used to build our search strategy using Boolean operators. The search strategy for Science Direct was ‘((Eustachian tube) OR (eustachian tube dysfunction)) AND ((BDET) OR (eustachian tube Balloon dilatation) OR (tuboplasty)))’. For PubMed, we used ‘((((Eustachian tube) OR (eustachian tube dysfunction)) AND (BDET)) OR (eustachian tube Balloon dilatation)) OR (tuboplasty)’. While for The Cochrane Library we used ‘("eustachian tube"):ti,ab,kw OR ("eustachian tube dysfunction"):ti,ab,kw AND (BDET):ti,ab,kw OR (Eustachian tube balloon dilatation):ti,ab,kw OR (Tuboplasty):ti,ab,kw’. The only search filter used was ‘research article’ for Science Direct. No date restrictions were used for the search in any of the databases. Additionally, we searched the reference lists of the included articles to ensure we did not miss any relevant studies.

Eligibility Criteria and Outcomes

We included studies conducted in the pediatric population (i.e., age <18 years) with middle ear fluid present for >3 months or tympanic membrane retraction, along with hearing loss. Studies were included if patients indicated tympanostomy tube (TT), and with no improvement after conventional therapy, which included either a history of adenoidectomy or a minimum of 1 TT insertion. The main intervention was BDET. We included cohorts, either prospective or retrospective, and trials. For a study to be included, it also had to report on at least one of the study outcomes.

Studies were excluded if they were conducted on patients aged 18 years or older or included all populations, with no separate data available for cases aged under 18 years. We excluded patients with craniofacial defects, cleft palate, Down syndrome, immunodeficiency, cystic fibrosis, malignant disease, and neuromuscular disorders, as these increase susceptibility to recurrent infections. We also excluded non-English studies and studies that did not report on the desired postoperative outcomes. Study designs other than RCTs and cohorts were also excluded.

The primary outcome of this study was improvement in tympanogram type postoperatively, defined as a change from type B or C to type A, and the absence of worsening from type A to type B or C. The secondary outcomes included risk of failure and complications. Failure was defined as no improvement in type B and C tympanograms, worsening of type A tympanograms, or recurrence of infection or effusion, along with the need for additional procedures.

Screening, Selection, and Data Extraction

Our search strategy on different databases yielded several articles that were initially searched for duplicates. After removing duplicates, reviewers independently assessed the relevance of the articles based on the title and abstract. The articles that passed this stage were searched for full texts, and studies with available full texts were then screened against our selection criteria. Articles that fully met the eligibility criteria were included in the final analysis. External help was used with methodology and synthesis. The whole process was carried out by two reviewers (ZZ and DC) independently, and any confusion regarding the inclusion or exclusion of an article was addressed by another reviewer (ZA), who independently made the selection decision.

The data from the included studies were extracted into an Excel sheet (Microsoft Corporation, Redmond, Washington, United States) with organized headings for each data item to be extracted. They included the study ID, design, age, gender, groups (intervention vs. control, if any), preoperative air-bone gap, preoperative tympanogram type, history of previous surgery, duration of follow-up, and key outcomes. This process was carried out by one reviewer and then rechecked by another reviewer.

Quality Assessment and Data Synthesis

The quality of the cohorts was assessed using the Newcastle Ottawa Scale (NOS) [15]. It consists of three domains: the selection domain, with four items and a maximum of 4 points; the comparability domain, with one item and a maximum of 2 points; and the outcome domain, with three items and a maximum of 3 points. The studies were graded as good quality if the score was ≥ 8; as fair quality if the score was ≥ 6 and < 8; and as poor quality if the score was < 6.

A single-arm meta-analysis was conducted because most of the included studies lacked a comparator. Proportions were pooled to estimate outcome prevalence using the inverse-variance method and the random-effects model and Freeman-Tukey Double arcsine transformation, with 95% confidence intervals by putting data into MetaAnalysisOnline.com software for meta-analysis [16]. Statistical heterogeneity was calculated using I2, with values>40% considered significant per the Cochrane Handbook [17]. I2 was considered low if it was below 25%, mild if 26-50%, moderate if 51-75%, and high if>75%. A forest plot was used to represent outcomes of the meta-analysis. Publication bias was assessed using the funnel plot and Egger's test. A sensitivity analysis was also conducted using the leave-one-out method, based on the quality of the studies assessed using NOS. Subgroup analysis could not be conducted due to the limited data available. Additionally, complications were presented using a table with frequencies and percentages.

Results

**Figure 1:**
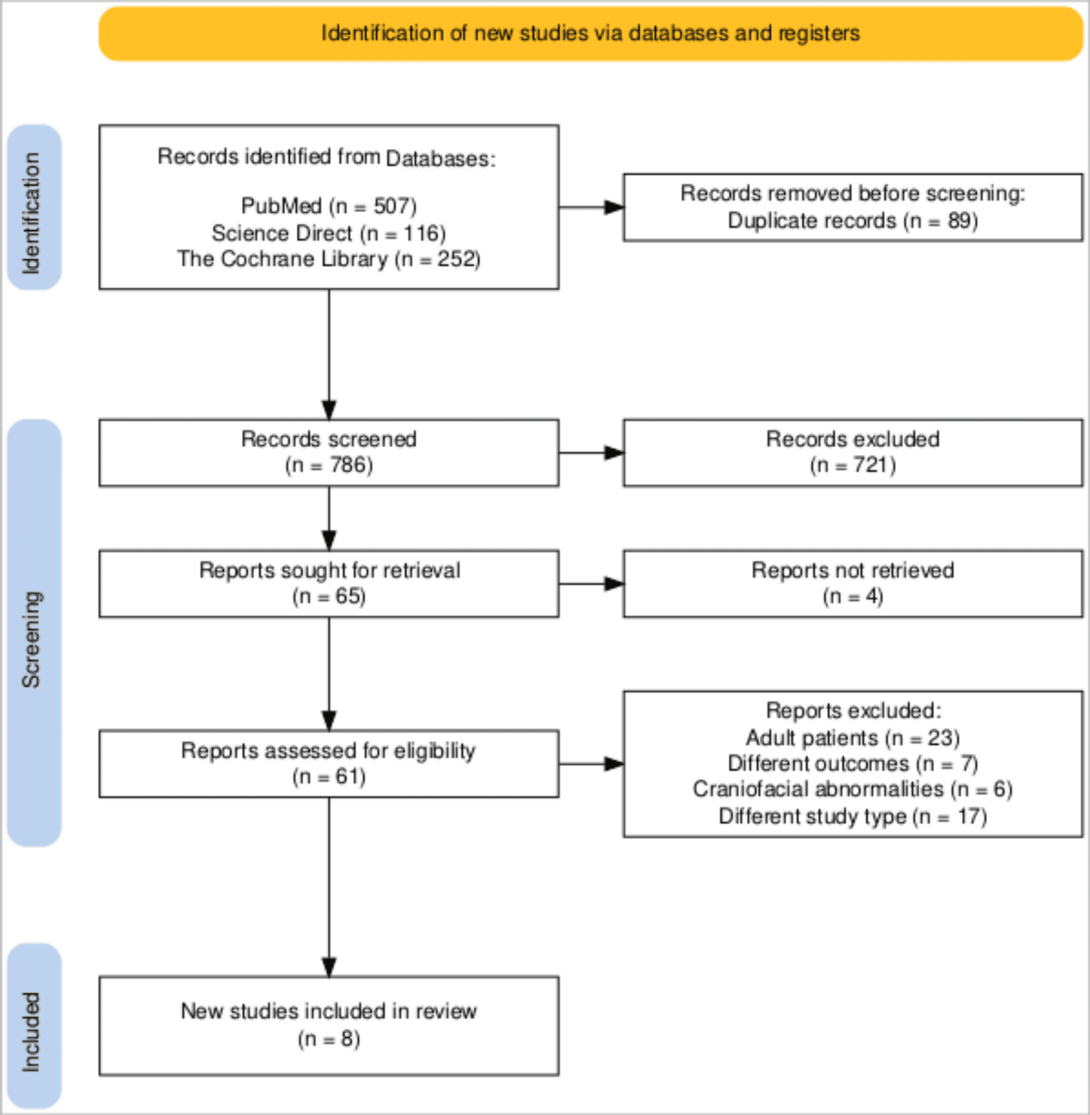
PRISMA flow diagram of study selection PRISMA: Preferred Reporting Items for Systematic reviews and Meta-Analyses

Our initial search across the three main databases yielded about 875 articles, of which 89 were classified as duplicates. Of the remaining articles that underwent title and abstract screening, 721 were excluded for irrelevance to the studies. The full text of four articles could not be found, so selection criteria were applied to the 61 remaining articles. Of these, 53 articles could not fulfil the selection criteria, and only eight articles were included in our final analysis [18,19,20,21,22,23,24,13]. The search of the reference lists yielded no relevant articles. The screening and selection process, along with the reasons for excluding articles, has been summarized in the PRISMA flowchart in Figure 1.

Characteristics of Studies

This review comprised eight retrospective cohorts, with almost all studies published within the last six years, except the study by Leichtle et al. (2017) [21]. This systematic review comprised 652 patients and 1214 ears. Five of the eight studies had no comparators [13,18,20,21,23], while the remaining three studies used ventilation tube (VT) (33.3%) and tympanostomy tube (TT) (66.4) as the comparators [19,22,24]. BDET was the primary intervention across all studies. Only two studies reported on the preoperative air-bone gap for BDET [19,22]. All studies had patients under the age of 18. In the context of previous surgeries, adenoidectomy was the most common procedure (7/11), followed by VT (2/11), tympanostomy (1/11), and tympanoplasty (1/11), with 11 being the combined number of surgeries from each study. Preoperative tympanogram results showed that type B was the most prevalent (63.4%), followed by type C (22.54) and type A (14.06%). The follow-up duration varied across studies, ranging from one month to seven years. Seven studies reported postoperative tympanogram results [13,18,19,21-24], and only four reported a risk of failure. The characteristics of the included studies are summarized in Table 1.

**Table 1:** Characteristics of included studies BDET: balloon dilatation of the eustachian tube; VT: ventilation tube; TT: tympanostomy tube

Study (author, year)	Design	Age	Ears/Patients, n	Intervention/Control	Air Bone Gap BDET	Tympanogram Type A/B/C, n	Previous Surgeries	Follow-Up Duration
Tisch et al., 2020 [18]	Retrospective Cohort	Mean (SD): 9.1 (2.6)	299/167	BDET/-	-	34/155/69	Adenoidectomy, Tympanoplasty, VT	Median: 2.6 months (95% CI: 0.3–16.1)
Demir and Batman, 2020 [19]	Retrospective Cohort	Mean (SD): 7 (2)	115/62	BDET/VT	Mean (SD): 27.6 (8.2) dB	0/115/0	Adenoidectomy	Mean: 14.4 months (range: 13–16)
Howard et al., 2021 [20]	Retrospective Cohort	Mean (SD): 12.4 (3.2)	81/43	BDET/-	-	-	-	30 days
Leichtle et al., 2017 [21]	Retrospective Cohort	mean 7 (Range, 3 - 15)	97/52	BDET/-	-	14/54/15	Adenoidectomy, VT	1 year
Toivonen et al., 2021 [22]	Retrospective Cohort	mean 12.5 (Range, 7-17)	46/26	BDET/TT	Mean (SD): 17.5 (11.9)	18/11/11	Tympanostomy, Adenoidectomy	Mean: 2.3 years (1.1)
Mukerji et al., 2024 [23]	Retrospective Cohort	mean 13.3 (Range, 8-18)	85/43	BDET/-	-	18/20/13	Adenoidectomy	Range: 1-28 months
Gurberg et al., 2024 [24]	Retrospective Cohort	Median: 7.5 (IQR 5-9) years	66/40	BDET/TT	-	0/42/24	Adenoidectomy	Mean: 6.7 years (2.6)
Liu et al., 2025 [13]	Retrospective Cohort	Mean (SD): 11 (3.69)	425/219	BDET/-	-	18/63/31	Adenoidectomy	Mean: 4.9 years (3.6)

Quality of Cohorts

The quality of the cohorts assessed using NOS showed that the majority (5/8) of studies had fair quality, with all failing to report a clear control cohort, except Demir 2020 et al. [19], and failing to adjust for related confounders. Only two studies were graded as high quality, scoring 9/9 [22,24]. In contrast, the study by Leichtle et al. was considered of poor quality due to missing data, no adjustment for confounders, and no clear reporting of the non-exposed cohort [21]. The NOS scoring summary is shown in Table 2.

**Table 2:** Quality assessment of included studies using the Newcastle-Ottawa Scale

Study	Selection (maximum score=4)	Comparability (maximum score=2)	Outcome (maximum score=3)	Total (maximum score=9)	Quality
Tisch et al., 2020 [18]	3	0	3	6	Fair
Demir et al., 2020 [19]	4	0	3	7	Fair
Howard et al., 2020 [20]	3	0	3	6	Fair
Leichtle et al., 2017 [21]	3	0	2	5	Poor
Toivonen et al., 2021 [22]	4	2	3	9	High
Gurberg et al., 2024 [24]	4	2	3	9	High
Mukerji et al., 2024 [23]	3	0	3	6	Fair
Liu et al., 2025 [13]	3	0	3	6	Fair

Primary Outcome

**Figure 2:**
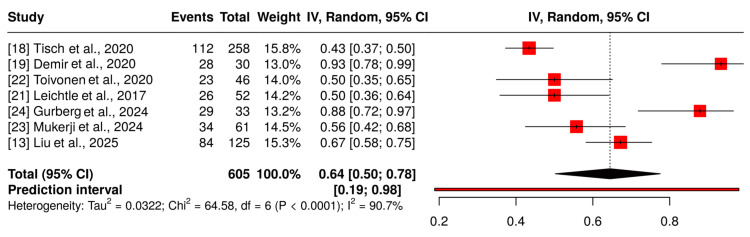
Forest plot showing improvement in tympanogram after BDET Random-effects model with 95% confidence intervals is presented. BDET: balloon dilatation of the eustachian tube References:  [13,18-21,23,24]

We assessed the efficacy of BDET by pooling data from cases that improved on tympanogram from type B and C to A, and from type A cases that showed no worsening. Our analysis of data from seven cohorts showed that 64% of cases undergoing BDET improved on tympanogram, with the correct percentage lying between 50% and 78%. The heterogeneity of this outcome was high (64%; 95% CI 50-78; I2 = 90.7%). We also conducted sensitivity analyses by lengthening one study step at a time and by excluding low-quality studies. However, the heterogeneity remained the same. The results of this outcome are shown in Figure 2.

**Figure 3:**
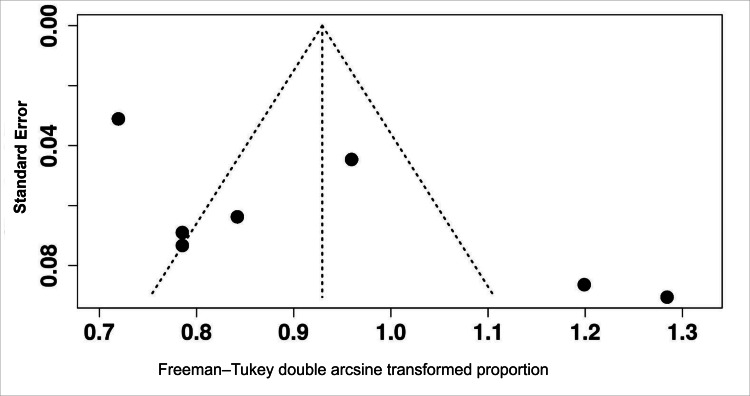
Funnel plot assessing publication bias for tympanogram improvement Funnel plot demonstrating the distribution of included studies assessing tympanogram improvement following BDET. The symmetrical distribution of studies around the pooled estimate suggests no significant publication bias, which is further supported by Egger’s test (p = 0.084). BDET: balloon dilatation of the eustachian tube References: [13,18-21,23,24]

Publication bias was assessed using funnel plot and Egger's test, showing no publication bias (Intercept 5.62; 95%CI 0.51 - 10.73, t = 2.155, p = 0.084) as shown in Figure 3.

Secondary Outcome

**Figure 4:**
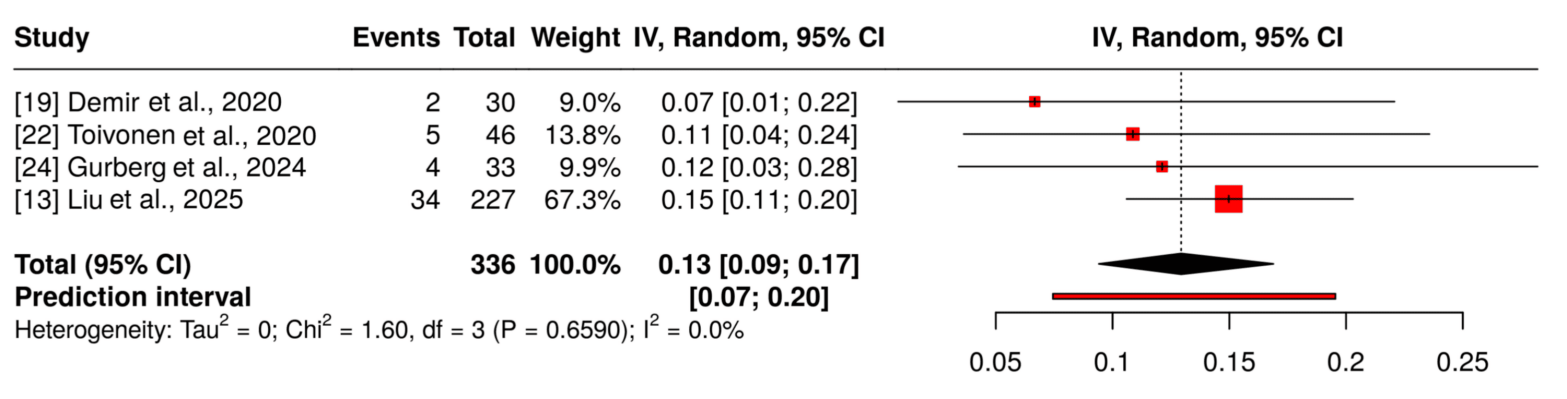
Forest plot showing risk of failure after BDET Failure was defined as no improvement, worsening tympanogram, or recurrence of symptoms. BDET: balloon dilatation of the eustachian tube References: [13,19,22,24]

We also assessed the risk of failure with the use of BDET. The majority of the studies did not have a comparator, so we pooled the outcome for BDET only. Only four of the included studies reported this outcome. Our analysis showed that of all the cases undergoing BDET, only 13% had an event of failure at different time points, and the associated statistical heterogeneity was zero (13%; 95% CI 1-17; I2 = 0%) with actual percentage falling between 1 and 17%. The results of this outcome are shown in Figure 4.

Complications

No major complications were observed with the BDET surgical procedure. However, we observed a total of 29 mild complications, with epistaxis and patulous eustachian tube being the most common. A list of observed complications is shown in Table 3.

**Table 3:** Reported complications following BDET All complications were minor and self-limiting. BDET: balloon dilatation of the eustachian tube

Complication	Frequency (Percentage)
Epistaxis	11 (37.9)
Patulous Eustachian Tube	7 (24.1)
Hemotympanum	5 (17.2)
Vertigo	2 (6.8)
Aborted Procedure	1 (3.5)
Nausea	1 (3.5)
Tinnitus	1 (3.5)
Otalgia	1 (3.5)

Discussion

This meta-analysis and systematic review assessed the effectiveness and safety of BDET in children with ETD. Eight studies (652 patients, 1214 ears) demonstrated a moderate improvement in middle ear ventilation with a pooled improvement rate of 64% (95% CI: 50-78%) in tympanogram outcomes. Although the observed improvement is comparable with other literature on the efficacy of BDET in recalcitrant ETD, the heterogeneity (I² = 90.7%) is high, indicating substantial variation between studies and limiting the consistency and reliability of the pooled estimate. Overall, these findings suggest that BDET may be a safe and potentially effective intervention in chronic or recalcitrant ETD in children [12,25]. The relatively small number of included studies reflects the limited availability of high-quality pediatric research in this area rather than deficiencies in the search strategy.

The observed improvement in tympanogram outcomes is likely attributable to the restoration of eustachian tube patency and improved regulation of middle ear pressure. BDET enhances middle ear ventilation by mechanically dilating the cartilaginous portion of the eustachian tube, thereby facilitating pressure equalization [26,27]. This mechanism is particularly relevant in pediatric populations, where anatomical and functional immaturity contribute to chronic dysfunction.

Notably, substantial heterogeneity was observed in the pooled analysis (I² = 90.7%), reflecting variability in study design, patient characteristics, prior interventions, and outcome reporting across the included studies. Differences in outcome definitions and follow-up durations may have further contributed to this inconsistency, underscoring the need for standardized assessment criteria in future research. This study provides an updated quantitative synthesis focused exclusively on pediatric populations, with pooled estimates of tympanogram improvement and failure rates. By restricting the analysis to children and applying a meta-analytic approach, our findings offer more precise and clinically relevant estimates, thereby strengthening the evidence base for pediatric practice.

In our analysis, BDET was identified as a safe procedure with no notable complications in the pediatric population. There were 29 minor, self-limiting complications, with epistaxis and a patulous eustachian tube most frequently. Other forms of complications, including hemotympanum, vertigo, otalgia, tinnitus, and nausea, were uncommon. The results are consistent with previous studies reporting low complication rates and positive safety outcomes [20,22,23,28]. These findings support the role of BDET as a minimally invasive intervention with a favorable safety profile in appropriately selected pediatric patients.

Nevertheless, the overall quality of evidence is low because most of the included studies are retrospective and lack comparator groups. This limits a direct comparison of BDET and traditional treatment procedures, including tympanostomy tube insertion, and introduces the risk of bias, including selection bias and confounding. These findings should be interpreted with caution due to the predominance of retrospective studies, lack of control groups, and substantial heterogeneity.

Limitations

This study has several limitations to its findings. Firstly, the majority of the studies were retrospective observational cohorts, which are susceptible to selection bias, recall bias, and confounding. Causal inferences cannot be as robust as those from randomized controlled trials. Second, most studies lacked comparison groups, necessitating a single-arm meta-analysis. This limits direct comparison of BDET with other traditional treatments, such as tympanostomy tubes or medical treatment. Third, the primary outcome was highly heterogeneous, and this heterogeneity should be justified by variations in patient characteristics, previous interventions, follow-up periods, and outcome definitions. Despite the sensitivity analyses, high heterogeneity was observed. Fourth, failure to consistently report the main variables, such as air-bone gap, symptom scores, and standardized definitions of failure, impeded subgroup analysis and a more in-depth quantitative synthesis. Additionally, subgroup analysis and meta-regression could not be performed due to the limited availability and inconsistent reporting of data across the included studies. Fifth, there were differences in the follow-up periods used across the studies, with some reporting only short-term outcomes. This limits the generalizability of BDET's long-term effectiveness and sustainability. Sixth, the small number of included studies, despite the lack of statistical significance in the publication bias, reduces the credibility of the funnel plot and Egger's test. Lastly, it did not include any studies in non-English, potentially introducing language bias and shaping the evidence base. Therefore, the current evidence remains insufficient to draw definitive conclusions regarding efficacy. These limitations highlight the need for cautious interpretation of findings and emphasize the importance of conducting high-quality randomized controlled trials in this field.

Future Directions

More studies are needed to overcome evidence gaps and elucidate the role of BDET in ETD management in children. Comparative studies between BDET and regular care, such as tympanostomy tube insertion, should be properly designed and their results compared to assess comparative effectiveness and long-term outcomes. There should also be standardised outcome measurements, such as standardised definitions of improvements in tympanograms, failure, and recurrence, to enhance comparability of the studies.

Long-term follow-up in future studies should also be included to assess the persistence of treatment effects and the risk of recurrence. Moreover, subgroup analyses by age, baseline disease severity, and a history of previous surgery would be effective in identifying the most receptive patient groups for BDET. Patient-reported outcomes, such as hearing-related quality of life and functional improvement, would provide a more detailed measure of clinical benefit. Lastly, the cost-effectiveness and timing of BDET as part of the treatment algorithm for pediatric ETD needed to be left as a subject for research.

## Conclusions

Overall, the findings of this meta-analysis suggest that BDET is a safe and potentially effective intervention in children with ETD, leading to improved tympanogram results and a low failure rate. The treatment seems to be associated with some minor complications. The majority of observational studies, however, are constrained by the lack of control groups and high heterogeneity. Therefore, there is a need to conduct well-structured randomized controlled trials with standardized outcome measures to determine their effectiveness and role in clinical care.
